# TRAJECTORIES OF ANXIETY SYMPTOMS DURING THE COVID-19 PANDEMIC AMONG OLDER ADULTS

**DOI:** 10.1093/geroni/igac059.1945

**Published:** 2022-12-20

**Authors:** Soyoung Choun, Dylan Lee, Maria Kurth, Hye Soo Lee, Heidi Igarashi, Carolyn Aldwin

**Affiliations:** Oregon State University, Corvallis, Oregon, United States; Touro University Nevada, Henderson, Nevada, United States; Oregon State University, Corvallis, Oregon, United States; Oregon State University, Corvallis, Oregon, United States; Oregon State University, Corvallis, Oregon, United States; Oregon State University, Corvallis, Oregon, United States

## Abstract

Older adults face particular challenges during the COVID-19 pandemic, including increased risk for morbidity and mortality (CDC, 2021). Social distancing and lockdown to prevent contagion may create social isolation and loneliness, adversely affecting mental and physical health. We examined anxiety symptom trajectories of older adults and identified risk and protective factors during the early months of the COVID-19 pandemic. We also examined how anxiety symptoms were associated with both between- and within-person variations in loneliness, social contacts, and physical problems. We sampled 247 older adults (Mage = 71.1, SD = 7.3, range = 51 - 95), who participated in eight weekly longitudinal online surveys from April 28 to June 23, 2020. Multilevel modeling analysis controlling for age, gender, marital status, and education showed that anxiety symptoms significantly decreased in the first few weeks, but then increased around week 6 of the study period. At the between-person level, individuals with higher levels of both loneliness and physical problems were at risk for experiencing higher levels of anxiety, but social contacts were not significant. Middle-aged participants reported higher anxiety symptoms than older participants. Women experienced higher anxiety symptoms than men. As a protective factor, individuals who were high in resilience had significantly lower anxiety symptoms over time, compared to those with low in resilience. In the within-person level, anxiety symptoms were positively coupled with both loneliness and physical symptoms over time. We conclude that lonely individuals and those in poor health were at greater risk of poor mental health during the pandemic.

